# Complete mitochondrial genome of the green-lipped mussel, *Perna canaliculus* (Mollusca: Mytiloidea), from long nanopore sequencing reads

**DOI:** 10.1080/23802359.2018.1437810

**Published:** 2018-02-09

**Authors:** Louis Ranjard, Thomas K. F. Wong, Carsten Külheim, Allen G. Rodrigo, Norman L. C. Ragg, Selina Patel, Brendon J. Dunphy

**Affiliations:** a Research School of Biology, ANU College of Science, The Australian National University, Canberra, Australia;; b Cawthron Institute, Nelson, New Zealand;; c School of Biological Sciences, University of Auckland, Auckland, New Zealand

**Keywords:** Mitochondrion, nanopore sequencing, bivalvia, *Perna canaliculus*

## Abstract

We describe here the first complete genome assembly of the New Zealand green-lipped mussel, *Perna canaliculus*, mitochondrion. The assembly was performed *de novo* from a mix of long nanopore sequencing reads and short sequencing reads. The genome is 16,005 bp long. Comparison to other Mytiloidea mitochondrial genomes indicates important gene rearrangements in this family.

The green-lipped mussel (*Perna canaliculus* Gmelin 1791, branded as ‘Greenshell™’ in aquaculture) is ecologically and economically important in New Zealand. Several mussels were collected on the west coast of the North Island of New Zealand. DNA extraction was performed on liquid nitrogen-homogenized samples following modified CTAB protocol (Winnepenninckx et al. 1993) and column extraction (Qiagen Inc., Valencia, CA). DNA extracted from 28 individuals was pooled and sequenced with an Oxford Nanopore Technologies MinION^TM^ sequencer. *De novo* genome assembly of the reads was performed using Canu (v1.5, Koren et al. 2017). A single long contig was identified to match the *P. canaliculus* mitochondrial *cox1* and *nd4* genes (Genbank DQ343591 and DQ343572). Subsequently, the sequencing reads aligning to this contig with high mapping quality (>30) were assembled using Unicycler (Wick et al. 2017) to obtain a circular genome reference.

One additional sample provided by the Cawthron Institute (Nelson, New Zealand) from an aquaculture long-line farm in Pelorus Sound (41°09′36.0″S 173°51′36.0″E, South Island, New Zealand), was analyzed for RNA sequencing on an Illumina HiSeq platform (Boyle 2016). The resulting ∼4.9 million short reads were aligned to the circular reference (mean coverage ∼37,987x for 99.86% of the reference) and the consensus sequence was constructed in Geneious (v10.2.3; Auckland, New Zealand). This step allowed us to build the mitochondrial genome for this specific individual (GenBank accession number MG766134).

The nucleotide composition of the 16,005 bp long mtDNA was 28.6% of A, 12.0% of C, 20.4% of G, and 39.0% of T for a GC content of 32.4%. Annotation was done in Geneious (v10.2.3; Auckland, New Zealand) using *P. viridis* as a reference (NC_018362) and the tRNA gene positions were confirmed with tRNAscan-SE (Lowe and Chan 2016). The circular genome contains 2 rRNA genes, 23 tRNA genes, and 13 protein-coding genes.

A total of 14 Mytiloidea species mitochondrial sequences could be retrieved at the time of the study: *Bathymodiolus platifrons* (NC_035421.1), *Brachidontes exustus* (NC_024882.1), *Limnoperna fortunei* (NC_028706.1), *Modiolus modiolus* (NC_033537.1), *M. philippinarum* (NC_035422.1), *Musculista senhousia* (NC_014590.1), *Mytilus californianus* (NC_015993.1), *M. chilensis* (NC_030633.1), *M. coruscus* (NC_024733.1), *M. edulis* (NC_006161.1), *M. galloprovincialis* (NC_006886.2), *M. trossulus* (NC_007687.1), *P. perna* (NC_026288.1) and *P. viridis* (NC_018362.1). Phylogenetic analysis was performed on concatenated protein-coding genes from all mitogenomes plus *Crassostrea gigas* (NC_001276.1): *atp6*, *cox1*, *cox2*, *cox3*, *cytb*, *nd1*, *nd2*, *nd3*, *nd4*, *nd4l*, *nd5*, *nd6*. Alignment was built for each gene independently with MUSCLE (Edgar 2004), sites were filtered with AliStat (Misof et al. 2014) and phylogenetic tree reconstructed with IQ-Tree (v1.5.4 Kalyaanamoorthy et al. 2017) and BEAST (v2.4.1 Bouckaert et al. 2014) resulting in identical topologies (Figure 1). The closest relative to *P. canaliculus* was *P. viridis*; however, Wood et al. (2007) reported a closest relationship to *P. perna.* Therefore, additional individual full genomes of *Perna* species are required to resolve their phylogeny. The gene order between *P. canaliculus* and *P. viridis* was compared using the R package genoPlotR (Guy et al. 2010) and showed the presence of several gene rearrangements in the mitochondrial genome (data not shown).

**Figure 1.**
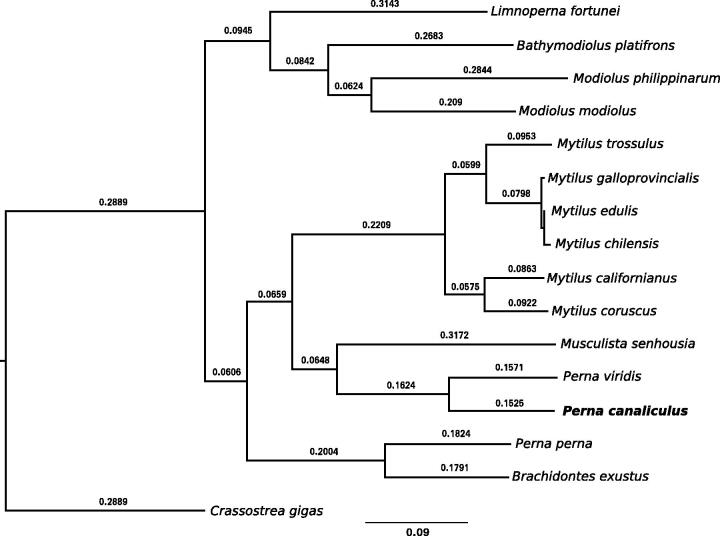
Maximum-likelihood phylogenetic tree of Mytiloidea family using concatenated mitochondrial protein-coding genes.
